# Health Care Professional Adherence to Breast Cancer Management Guidelines in Nigeria

**DOI:** 10.1001/jamanetworkopen.2024.59614

**Published:** 2025-02-12

**Authors:** Anya Romanoff, Olalekan Olasehinde, Kathleen Lynch, Sharif Folorunso, Oluwatosin Omoyiola, Betiku Omolade, Adeleye Omisore, Chukwuma Eze Okereke, Olayide Agodirin, Abubakar Bala Muhammad, Nuhu Ali, Omorodion Irowa, Nneka Sunday Nweke, Uchechukwu Emmanuel Nwokwu, Adewale Aderounmu, Funmilola Wuraola, Hannah L. Kalvin, Rivka Kahn, Grace Fitzgerald, Cristina Olcese, Alexia Iasonos, Victoria L. Mango, Jamie S. Ostroff, Rachel Vreeman, Benjamin O. Anderson, T. Peter Kingham, Olusegun Isaac Alatise

**Affiliations:** 1 Department of Global Health and Health System Design, Icahn School of Medicine, Mount Sinai, New York, New York; 2 Global Cancer Disparities Initiatives, Memorial Sloan Kettering Cancer Center, New York, New York; 3 Department of Surgery, Obafemi Awolowo University Teaching Hospitals Complex, Ile Ife, Nigeria; 4 Department of Psychiatry and Behavioral Science, Memorial Sloan Kettering Cancer Center, New York, New York; 5 Department of Social and Behavioral Sciences, New York University School of Global Public Health, New York; 6 Department of Radiology, Obafemi Awolowo University Teaching Hospitals Complex, Ile Ife, Nigeria; 7 Department of Pathology, Obafemi Awolowo University Teaching Hospitals Complex, Ile Ife, Nigeria; 8 Department of Surgery, Federal Medical Center Owo, Owo, Nigeria; 9 Department of Surgery, University of Ilorin and University of Ilorin Teaching Hospital, Ilorin, Nigeria; 10 Department of Surgery, Aminu Kano Teaching Hospital, Kano, Nigeria; 11 Department of Surgery, University of Maiduguri Teaching Hospital, Maiduguri, Nigeria; 12 Department of Surgery, University of Benin Teaching Hospital, Benin, Nigeria; 13 Department of Surgery, Alex Ekwueme Teaching Hospital Abakaliki, Abakaliki, Nigeria; 14 Federal Ministry of Health, Abuja, Nigeria; 15 Department of Epidemiology and Biostatistics, Memorial Sloan Kettering Cancer Center, New York, New York; 16 City Cancer Challenge (C/Can), Geneva, Switzerland; 17 Department of Surgery, University of Washington, Seattle; 18 Department of Global Health, University of Washington, Seattle

## Abstract

**Importance:**

In Nigeria, 80% of breast cancer is diagnosed at an advanced stage, when clinical management is complex and necessitates multidisciplinary collaboration. The Nigerian Federal Ministry of Health promotes multidisciplinary tumor board (MDT) discussions and endorses the National Comprehensive Cancer Network Harmonized Guidelines for Sub-Saharan Africa (Harmonized Guidelines) to standardize breast cancer care, but the extent of their clinical utilization is not well understood.

**Objective:**

To identify barriers to and facilitators of the standardization of breast cancer care in Nigeria as reflected by awareness and consultation of resource-adapted guidelines.

**Design, Setting, and Participants:**

For this survey study, health care professionals (HCPs) involved in breast cancer care in Nigeria were recruited electronically through medical societies and snowball sampling from November 1, 2023, to January 31, 2024.

**Exposure:**

Breast cancer care guidelines.

**Main Outcomes and Measures:**

Current breast cancer guideline awareness, adherence among specialties, and multidisciplinary management in Nigeria were evaluated. A multinational, multidisciplinary team developed and distributed an HCP questionnaire. Cochran-Armitage trend tests and logistic regression assessed associations with guideline awareness and use.

**Results:**

A total of 277 HCPs (median [IQR] age, 42 [38-48] years; 211 of 275 [77%] male), including 124 surgeons (45%), 59 radiologists (21%), 49 clinical oncologists (18%), and 45 pathologists (16%), responded to the survey. Of 274 HCPs, 253 (92%) believed that breast cancer guidelines contribute to better patient outcomes, and 100 of 195 (51%) routinely consulted guidelines. Of 273 HCPs, 103 (38%) had regular engagement in breast cancer MDT discussions. Only 93 of 277 HCPs (34%) were aware of resource-adapted Harmonized Guidelines; both guideline awareness and consultation increased with the frequency of MDT participation. Completing a cancer-related fellowship (OR, 14.56; 95% CI, 6.24-40.00; *P* < .001), clinical oncology specialization (OR vs surgery, 0.20 [95% CI, 0.09-0.42]; OR vs pathology, 0.05 [95% CI, 0.02-0.14]; OR vs radiology, 0.02 [95% CI, 0.01-0.06]; *P* < .001), devoting more time to breast health care delivery (OR, 1.04; 95% CI, 1.02-1.05; *P* < .001), and regular MDT engagement (OR for weekly vs none, 5.13; 95% CI, 1.69-17.35; *P* = .04) were associated with consulting guidelines regularly on univariable analysis. To improve guideline adherence, 230 of 277 HCPs (83%) reported needing regular MDT discussions.

**Conclusions and Relevance:**

In this survey of HCPs across Nigeria, half reported routinely consulting guidelines and only one-third were aware of the Harmonized Guidelines, which are resource adapted and endorsed by the Nigerian Federal Ministry of Health. Approximately one-third of HCPs reported engaging in regular breast cancer MDT discussions; guideline awareness and consultation increased as frequency of MDT participation increased. Opportunities for disseminating knowledge, such as MDT discussions and fellowship training, are essential to standardize breast cancer care delivery in Nigeria.

## Key Points

**Question:**

What are the barriers to and facilitators of the standardization of breast cancer care in Nigeria as measured by use of resource-adapted guidelines?

**Findings:**

In this survey study of 277 health care professionals in Nigeria, 38% reported routinely engaging in multidisciplinary tumor board discussions. Awareness of resource-adapted National Comprehensive Cancer Network Harmonized Guidelines for Sub-Saharan Africa and guideline consultation increased as the frequency of multidisciplinary tumor board discussions increased.

**Meaning:**

These findings suggest that access to regular multidisciplinary tumor board discussions has the potential to standardize delivery of breast cancer care in Nigeria by improving awareness and use of resource-adapted guidelines.

## Teaser

This survey study examines barriers to and facilitators of adherence to breast cancer care guidelines reported among health care professionals in Nigeria.

## Introduction

Breast cancer is the most common malignant neoplasm and the leading cause of cancer-related mortality in women worldwide. Western Africa has among the highest mortality rates from breast cancer: 22.6 per 100 000 females compared with 11.3 per 100 000 females in high-income countries.^1^ In Nigeria, a low- and middle-income country with a population of more than 200 million people, 80% of breast cancer is diagnosed at stage III or IV,^2^ when clinical management is complex and necessitates multidisciplinary collaboration.

Evidence-based, resource-stratified clinical practice guidelines were introduced by the Breast Health Global Initiative in 2006 to standardize breast cancer care and strengthen health care systems in resource-limited settings.^3^ Multiple iterations of these guidelines have since been published, but challenges persist in implementing them in routine clinical practice.^4,5,6^ To better tailor guidelines to the local clinical context, in 2017, experts from 12 Sub-Saharan African (SSA) nations (through the African Cancer Coalition), in partnership with the American Cancer Society and the National Comprehensive Cancer Network (NCCN), developed the NCCN Harmonized Guidelines for Sub-Saharan African (Harmonized Guidelines). These guidelines are adapted specifically to direct breast cancer management in SSA.^7,8^

The Nigerian Federal Ministry of Health (FMOH) endorses the Harmonized Guidelines for breast cancer care. In its National Strategic Cancer Control Plan, most recently updated for 2023 to 2027, the FMOH identified the key need “to increase human capacity development for healthcare personnel in cancer diagnosis and treatment.”^9^ The broad strategy proposed to accomplish this is to “improve healthcare providers’ knowledge on standards of care for effective treatment and quality cancer care.”^9^ Performance indicators to measure progress include the “number of comprehensive cancer centers in the country with a functional multi-disciplinary tumor board” and the “number of comprehensive cancer centers in the country that have adopted and implemented the updated treatment guideline for the management of cancer patients.”^9^ Initial steps were taken by the FMOH to integrate multidisciplinary tumor board (MDT) discussions into routine cancer care, as mandated by a circular distributed in 2021. The impact of this mandate on engagement in routine MDT discussions has not yet been evaluated on a national scale, with some evidence that the Harmonized Guidelines are not widely adopted in Nigeria.^10^ Further evaluation is necessary to elucidate barriers and facilitators influencing the integration of these guidelines into clinical practice. In line with FMOH priorities for cancer care, this study aimed to understand guideline awareness and consultation by surveying health care professionals (HCPs) involved in breast cancer management across Nigeria. Ensuing knowledge of barriers to and facilitators of the standardization of breast cancer care will serve as a basis for developing an implementation plan to improve breast cancer care delivery in Nigeria.

## Methods

### Study Design

To assess awareness, current practices, and barriers to guideline use in Nigeria, we conducted a survey study of breast cancer HCPs across various specialties practicing nationwide. Institutional review board approval for this research was obtained in the US and in Nigeria from Obafemi Awolowo University Teaching Hospitals Complex, Ile Ife, Nigeria; Icahn School of Medicine at Mount Sinai, New York, New York; and Memorial Sloan Kettering Cancer Center, New York, New York. This report follows the American Association for Public Opinion Research (AAPOR) reporting guideline for survey studies.

### Questionnaire Development

Use of breast cancer guidelines by a national sample of Nigerian HCPs was evaluated. A questionnaire was developed by a multinational, multidisciplinary team, guided by the well-established Consolidated Framework for Implementation Research (CFIR).^11^ The questionnaire (eAppendix in Supplement 1) consisted of 4 sections: HCP (provider) characteristics, institution characteristics, perceived barriers and facilitators to guideline adherence, and practice patterns based on relevant clinical scenarios. The guideline adherence section was informed by CFIR, which assesses determinants of implementation across 5 domains (the intervention, inner setting [practice], outer setting [system], individuals involved, and implementation process), emphasizing the organizational context. CFIR has been used to improve understanding of factors associated with health care delivery and can be modified for use in low- and middle-income countries.^12,13,14^ Clinical practice scenarios were tailored for the 4 types of clinical specialists (surgeons, clinical oncologists, radiologists, and pathologists) and are reported separately. The questionnaire was pilot tested among 8 HCPs in Nigeria (2 from each specialty). The qualitative methodologist (K.L.) engaged each respondent in cognitive debriefing interviews to assess questionnaire comprehension and content validity and to elicit specialty-specific feedback. The final questionnaire was modified based on HCP input.

### Study Participants

Physicians involved in breast cancer care in Nigeria were eligible for the study, with a focus on surgery, clinical oncology, radiology, and pathology—key specialties in multidisciplinary management. Surgeons or clinical oncologists lead breast cancer management, while radiologists and pathologists play vital roles in diagnosis and MDT success. Participants included consultants (attending physicians) and senior registrars (trainees who have significant decision-making responsibilities in the Nigerian health care system). Exclusion criteria were junior registrars/residents and HCPs not practicing in listed specialty areas. According to national association platforms and team estimates, at the time of this research, Nigeria had approximately 90 clinical oncologists, 264 radiologists, 250 pathologists, and 300 consultant surgeons.

HCPs were recruited electronically through national and international medical association membership lists and snowball sampling from November 1, 2023, through January 31, 2024. The questionnaire was distributed by email and electronic messaging platforms and administered through Research Electronic Data Capture (REDCap). Informed consent was obtained through REDCap. Responses were anonymous, with each participant assigned a unique identification number and instructed to complete the survey once. All participants were of African ancestry due to the study taking place in Nigeria.

### Statistical Analysis

Descriptive statistics were calculated for each item as appropriate (numbers and percentages for categorical variables and medians and IQRs for continuous variables). We used a 5-point Likert scale item to assess guideline use, with response ranging from “never” to “always.” Consulting guidelines regularly was defined as responding “most of the time” or “always” to the question, “How often do you consult breast cancer guidelines in your clinical practice?” A 2-sided Cochran-Armitage trend test was performed to assess whether MDT participation frequency was associated with awareness of the Harmonized Guidelines or consultation of the guidelines. No weighting was used. Univariable logistic regression was used to assess the association between demographic and institutional factors of interest and consulting breast cancer guidelines regularly. Factors significant on univariable analysis were incorporated into a multivariable logistic regression analysis. Two-sided *P* < .05 was considered significant. All analyses were conducted using R, version 4.2.3 (R Foundation).^15^

## Results

### HCP and Institution Characteristics

This nationwide study included 277 participants (median [IQR] age, 42 [38-48] years; 211 [77%] male and 64 [23%] female of 275 with known sex), of whom 124 were surgeons (45%), 59 were radiologists (21%), 49 were clinical oncologists (18%), and 45 were pathologists (16%). HCPs had a median of 7 (IQR 3-12) years in practice and estimated that they devoted a median of 30% (IQR 20%-50%) of their practice to breast health care. A total of 217 of 274 (79%) practiced in a teaching hospital and 196 of 272 (72%) in an urban environment. HCPs from all geopolitical zones of Nigeria participated, with the largest proportion practicing in Southwest Nigeria (108 of 269 [40%]). Characteristics of the HCPs and institutions are detailed in Table 1.

**Table 1.** Health Care Practitioner and Institutional Characteristics

Characteristic	No. (%)^a^ (N = 277)
Age, median (IQR), y	42 (38-48)
Sex	
Male	211 (77)
Female	64 (23)
Missing	2
Profession	
Surgeon	124 (45)
Radiologist	59 (21)
Clinical oncologist	49 (18)
Pathologist	45 (16)
Professional status	
Consultant/attending	172 (62)
Senior registrar/resident	105 (38)
Length of practice after training, median (IQR), y	7 (3-12)
Completed fellowship in oncology	
No	232 (85)
Yes	40 (15)
<3 mo, No./total No. (%)	1/40 (2)
3-12 mo, No./total No. (%)	9/40 (22)
>12 mo, No./total No. (%)	30/40 (75)
Missing	5
Estimated total practice time devoted to breast health care, median (IQR), %	30 (20-50)
Research engagement	
None	68 (25)
Some	152 (57)
A lot	49 (18)
Missing	8
Facility type	
University teaching hospital	217 (79)
Federal medical center	25 (9)
Other (state or private specialist hospital or general hospital)	32 (12)
Missing	3
Geopolitical zone	
Southwest	108 (40)
South-South	41 (15)
Northcentral	38 (14)
Northwest	38 (14)
Southeast	23 (9)
Northeast	21 (8)
Missing	8
Facility location	
City	196 (72)
Town or semidense area	66 (24)
Rural	10 (4)
Missing	5
Approximate No. of inpatient beds in practice facility, median (IQR)	500 (280-800)
Driving distance from practice facility to next referral center, h	
<1	107 (43)
1-3	118 (48)
>3	23 (9)
Missing	29

^a^ Unless otherwise indicated. Missing values are excluded from the calculation of percentages.

### Inner Setting (Practice)

Among 273 HCPs with available data, 103 (38%) reported routinely engaging in MDT discussions (defined as weekly, biweekly, or monthly) regarding patients with breast cancer (eTable 1 in Supplement 1); 139 (51%) indicated that MDT discussions were held on a case-by-case basis. Only 52 (19%) reported weekly or biweekly MDT discussions. HCPs were asked about their workflow processes and receptivity to change (eTable 2 in Supplement 1). A total of 239 HCPs (86%) reported receiving some form of performance feedback about the care they provide. Feedback was mostly informal (152 [55%]), with only 45 participants (16%) receiving formal performance reviews from their department or institution, 26 (9.4%) receiving this information from written reports, and 9 (3.2%) from targeted metrics. A total of 147 of 244 HCPs (60%) had an institutional breast cancer patient database or registry at their institution, and 61 of 273 (22%) had regular access to an electronic data system to track patients. Although 164 of 272 HCPs (60%) reported the ability to try innovative practices, 63 (23%) felt supported by their organization in such efforts. A total of 158 of 271 HCPs (58%) thought their organization’s leadership would be moderately to extremely receptive to implementing structured breast cancer care programs to promote guideline adherence.

### Outer Setting (System)

HCPs reported learning about practice-changing innovations primarily through personal reading of the medical literature (224 [81%]) and colleague interactions (212 [77%]). The most common modalities for collegial discussions of complex clinical cases were departmental meetings (220 [79%]) and informal discussions (186 [67%]). Only 114 of 272 (42%) reported networking with other HCPs in similar professions or positions outside their clinical practice setting.

### Intervention Characteristics (Breast Cancer Guidelines)

Out of 274 HCPs, 253 (92%) reported agreement that breast cancer guidelines contribute to better patient outcomes (Table 2). Even though 206 (75%) were aware of established guidelines for breast cancer care, only 100 of 195 (51%) routinely consulted these in practice. The NCCN guidelines were most cited, but only 93 HCPs (34%) were aware of the FMOH-endorsed Harmonized Guidelines. The majority of HCPs accessed guidelines online (156 of 277 [56%]). Insufficient resources were reported by 188 of 274 (69%) as the reason for not routinely providing guideline-adherent care, and 122 of 257 (47%) regularly adjusted clinical recommendations based on patients’ capacity to adhere.

**Table 2.** Awareness and Use of Breast Cancer Management Guidelines

Measure	No. (%) of health care practitioners (N = 277)
Are you aware of any established national or international guidelines for breast cancer care? (yes)	206 (75)
NCCN–any	167 (60)
American Society of Clinical Oncology	142 (51)
NCCN Harmonized Guidelines for Sub-Saharan Africa	93 (34)
European Society of Medical Oncology	87 (31)
American Society for Radiation Oncology	59 (21)
NCCN resource-stratified guidelines	51 (18)
Breast Health Global Initiative	50 (18)
Tailored institutional guidelines	27 (10)
Cancer Care Ontario	15 (5)
Saint Gallen International Consensus Conference	10 (4)
How often do you consult breast cancer guidelines in your clinical practice?	
Never	3 (2)
Rarely	21 (11)
Sometimes	71 (36)
Most of the time	68 (35)
Always	32 (16)
Missing	82
How do you access breast cancer guidelines?	
Online	156 (56)
Mobile application (app)	77 (28)
Paper copy	22 (8)
To ensure the care I am providing is adherent to breast cancer guidelines, I would need these additional tools and/or resources:	
Regular structured multidisciplinary discussions about patient care	230 (83)
Guidelines tailored to my practice setting	205 (74)
Additional specialized education and training in breast cancer care	195 (70)
Infrastructure for communication among practitioners (consultation or bidirectional feedback)	186 (67)
Infrastructure for patient referrals and navigation through the health care system	185 (67)
Physically accessible guidelines	164 (59)
I believe breast cancer guidelines are applicable to the patient care setting in which I practice. (n = 271)	
Strongly disagree	17 (6)
Disagree	5 (2)
Neither agree nor disagree	10 (4)
Agree	104 (38)
Strongly agree	135 (50)
I think breast cancer guidelines contribute to better patient outcomes. (n = 274)	
Strongly disagree	14 (5)
Disagree	2 (1)
Neither agree nor disagree	5 (2)
Agree	85 (31)
Strongly agree	168 (61)
I think breast cancer guidelines are too complex to be useful for helping me make choices about patient care. (n = 273)	
Strongly disagree	79 (29)
Disagree	133 (49)
Neither agree nor disagree	32 (12)
Agree	19 (7)
Strongly agree	10 (4)
In my current practice, I have sufficient resources to provide care adherent to breast cancer guidelines. (n = 274)	
Strongly disagree	33 (12)
Disagree	92 (34)
Neither agree nor disagree	63 (23)
Agree	66 (24)
Strongly agree	20 (7)
How often do you adjust your clinical recommendations based on whether the patient will be able to follow them? (n = 257)	
Never	5 (2)
Rarely	26 (10)
Sometimes	104 (40)
Most of the time	83 (32)
Always	39 (15)

Abbreviation: NCCN, National Comprehensive Cancer Center.

Access to regular MDT discussions was the most frequent response selected to promote delivery of guideline-adherent care (230 [83%]). Other recommendations were for more tailored guidelines (205 [74%]), additional education or training in breast cancer care (195 [70%]), infrastructure for communication among practitioners (186 [67%]), and patient navigation programs (185 [67%]) (Table 2).

### Factors Associated With Consultation of Guidelines

**Figure 1.**
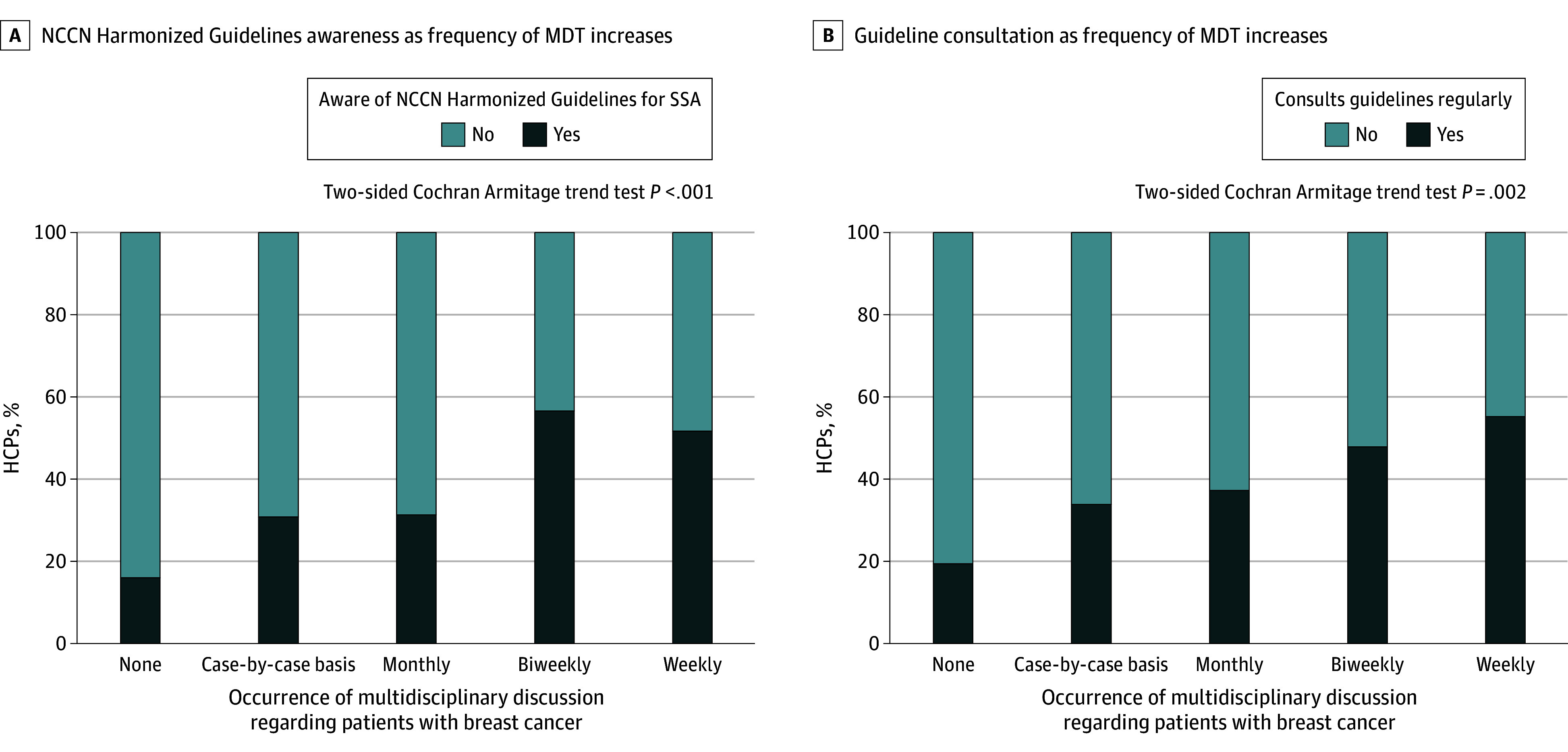
Associations of Guideline Awareness and Consultation With Multidisciplinary Tumor Board (MDT) Discussion Frequency HCP indicates health care practitioner; NCCN, National Comprehensive Cancer Network; SSA, Sub-Saharan Africa.

Increased frequency in MDT participation was associated with consulting guidelines more regularly (none: 6 of 31 [19%]; case by case: 47 of 139 [34%]; monthly: 19 of 51 [37%]; biweekly: 11 of 23 [48%]; and weekly: 16 of 29 [55%]; *P* = .002 for trend) (Figure 1). HCPs who engaged in MDT discussions more frequently were also more likely to have an increased awareness of the Harmonized Guidelines (none: 5 of 31 [16%]; case by case: 43 of 139 [31%]; monthly: 16 of 51 [31%]; biweekly: 13 of 23 [57%]; and weekly: 15 of 29 [52%]; *P* < .001 for trend). The most notable benefits were observed with weekly or biweekly sessions. On univariable analysis (Table 3), compared with those who did not engage in MDT discussions, respondents who engaged in weekly MDT discussions were more likely to consult guidelines regularly (OR, 5.13; 95% CI, 1.69-17.35; *P* = .04). Clinical oncologists were more likely than surgeons (OR, 0.20; 95% CI, 0.09-0.42), radiologists (OR, 0.02; 95% CI, 0.01-0.06), and pathologists (OR, 0.05, 95% CI, 0.02-0.14) to consult guidelines regularly (*P* < .001). Health care practitioners who had completed a cancer-related fellowship (vs those who had not) were more likely to consult guidelines regularly (OR, 14.56; 95% CI, 6.24-40.00; *P* < .001), as were HCPs who devoted more time to breast health care delivery (OR, 1.04; 95% CI, 1.02-1.05; *P* < .001). In the multivariable model (Table 3; eFigure in Supplement 1), profession, completing a fellowship in cancer care, and estimated percentage of practice devoted to breast health care remained significantly associated with consulting guidelines regularly.

**Table 3.** Health Care Professional and Institutional Factors Associated With Regular Guideline Consultation

Characteristic	Univariable		Multivariable	
	OR (95% CI)	*P* value	OR (95% CI)	*P* value
Age	0.98 (0.95-1.02)	.30	NA	NA
Sex	1.09 (0.60-1.93)	.80	NA	NA
Profession				
Clinical oncologist	1.00 [Reference]	<.001	1.00 [Reference]	<.001
Surgeon	0.20 (0.09-0.42)		0.60 (0.23-1.54)	
Pathologist	0.05 (0.02-0.14)		0.16 (0.05-0.51)	
Radiologist	0.02 (0.01-0.06)		0.07 (0.02-0.26)	
Professional status	0.94 (0.56-1.56)	.80	NA	NA
Length of practice after training	0.98 (0.94-1.01)	.20	NA	NA
Completed fellowship in cancer care				
No	1.00 [Reference]	<.001	1.00 [Reference]	<.001
Yes	14.56 (6.24-40.00)		5.91 (2.03-19.42)	
Estimated percentage of total practice devoted to breast health care	1.04 (1.02-1.05)	<.001	1.03 (1.01-1.05)^a^	<.001
Engage in multidisciplinary discussion				
None	1.00 [Reference]	.04	1.00 [Reference]	.80
Weekly	5.13 (1.69-17.35)		1.60 (0.39-6.80)	
Biweekly	3.82 (1.17-13.54)		1.89 (0.50-7.56)	
Monthly	2.47 (0.90-7.63)		2.00 (0.61-7.17)	
Case-by-case basis	2.13 (0.86-6.05)		1.81 (0.64-5.72)	
Research engagement				
None	1.00 [Reference]	.30	NA	NA
Some	1.59 (0.86-3.02)		NA	
A lot	1.63 (0.75-3.59)		NA	
Facility type				
University teaching hospital	1.00 [Reference]	.70	NA	NA
Federal medical center	1.46 (0.62-3.36)		NA	
Other (state or private specialist hospital or general hospital)	0.97 (0.43-2.09)		NA	
Facility location				
City	1.00 [Reference]	.40	NA	NA
Town or semidense area	1.27 (0.72-2.25)		NA	
Rural	0.46 (0.07-1.90)		NA	
Approximate No. of inpatient beds in practice facility	1.00 (0.96-1.04)	>.99	NA	NA
Driving distance from practice facility to next referral center, h				
<1	1.00 [Reference]	.80	NA	NA
1-3	0.92 (0.54-1.59)		NA	
>3	1.03 (0.40-2.58)		NA	

Abbreviations: NA, not applicable; OR, odds ratio.

^a^ For every 1% increase in total practice time devoted to breast health care, health care professionals were 3% more likely to consult guidelines regularly.

## Discussion

Despite the overwhelming acknowledgment of the importance of guidelines, this study found that only half of Nigerian HCPs routinely consulted breast cancer guidelines, and only one-third were aware of the FMOH-endorsed Harmonized Guidelines, highlighting the need for clinician support interventions. This research found that MDT participation frequency was associated with awareness and use of breast cancer clinical practice guidelines, suggesting that increasing access to MDT discussions could close the gap between FMOH recommendations and current practices. These results appear to be mediated by profession, completion of a fellowship in cancer care, and estimated percentage of practice time devoted to breast health care. Knowledge, training, and experience in breast cancer care delivery through fellowship training, practice time devoted to breast cancer care, or weekly or biweekly MDT participation have potential to improve guideline use and breast cancer care delivery. Although specialty, training, and breast cancer clinical volume are the main factors associated with guideline awareness and use in Nigeria, MDT access may offer a more easily modifiable target and pragmatic approach to begin to address these issues, as reported by HCPs themselves.

Our findings align with *The Lancet Oncology* Commission on Cancer in SSA’s Call to Action^16^ to improve cancer care delivery in the region by addressing several systemic limitations. These action items include prioritizing cancer care that is delivered by multidisciplinary teams and in line with national guidelines, improving access to data systems for patient tracking and progress monitoring, expanding universal health coverage and integrating cancer care into insurance schemes, and investing in education, training, and workforce retention.

### Barriers to and Facilitators of Guideline Adherence

#### Guideline Awareness

This study highlights limited awareness of the FMOH-endorsed Harmonized Guidelines as a key barrier to their use in Nigeria. More than two-thirds of HCPs reported insufficient resources to routinely deliver guideline-adherent care. However, most HCPs were not aware that resource-adapted guidelines for SSA exist. In a prior survey of 109 clinical and surgical oncologists in Nigeria, Adegboyega et al^10^ found similarly low uptake of the Harmonized Guidelines, with resource limitations cited as the most influential barrier. Our current study confirms these findings and expands them to other clinical specialties involved in breast cancer care. Limited awareness of resource-adapted guidelines has also been demonstrated in other SSA contexts. Most Ethiopian HCPs who treat hematologic cancers reported using guidelines, but only 23% used the Harmonized Guidelines.^17^ Increasing awareness of the Harmonized Guidelines through MDT discussions could be a first step to improve HCPs’ delivery of guideline-adherent care with available resources.

#### Siloed Care Delivery

Approximately one-third of survey respondents reported engaging in routine MDT discussions, fewer received formal performance feedback about the care they provide, and even fewer had regular access to an electronic data system to track patients. Most HCPs learned about innovation in the field through their own initiatives and without formal input on standardized care. Developing a care plan for a patient with breast cancer is complex and ever-evolving and necessitates multidisciplinary intervention, especially in a setting where most disease is locally advanced at diagnosis. Routine MDT discussions can provide a forum for delivering education in breast cancer care and developing resource-appropriate patient care plans. In addition, MDT discussions can provide a mechanism for improving quality and providing feedback.^18^

Development and engagement of MDT discussions requires financial investment, trained leadership, a physical space, and protected time.^19,20^ Most HCPs in Nigeria are generalists who treat a variety of disease entities and have limited time and bandwidth for additional commitments. Although MDT discussions are an FMOH priority,^9^ additional infrastructure is required to realize this plan in most institutions. Implementation science frameworks, particularly those focusing on behavior change, are well suited to address the barriers uncovered here and begin to develop context-adapted solutions.^21^ Telemedicine can be leveraged as a practical solution and valuable tool for overcoming geographic and logistical barriers to regular MDT engagement. For example, Project ECHO (Extension for Community Healthcare Outcomes) is a structured, case-based clinical education delivery platform that has been used for breast cancer care discussions, including in SSA.^22^

#### Workforce Limitations

Clinical oncologists were more likely to consult guidelines, possibly because they receive more cancer care training than the other HCP types. This finding is substantiated by the better adherence also reported among those who completed oncology fellowships and devote more of their practice to breast cancer care. There is a critical shortage of the oncology workforce in SSA, with a mean of 6.4 clinical oncologists per country.^16^ Developing greater access to oncology fellowship training in Nigeria is currently under way.^23^ Until workforce shortages improve, MDT discussions can provide opportunities for sharing knowledge across HCPs to address differences among specialties.

#### Practical Considerations in Guideline Applicability

Although international guidelines have been modified for resource-constrained settings (eg, the Harmonized Guidelines), limitations remain in HCPs’ ability to deliver care that adheres to these guidelines because of practical challenges. For example, according to guidelines, known receptor status is a prerequisite for treatment planning. However, limited ability to perform immunohistochemistry means that results are unavailable or unreliable in some settings.^24^ In addition, according to the Harmonized Guidelines, breast imaging with diagnostic bilateral mammography is an essential component of the workup for invasive, noninflammatory, nonmetastatic breast cancer.^25^ However, in Nigeria, where the mean breast tumor size is 10.5 cm^2^ and most cancer care is paid for by patients out of pocket, diagnostic bilateral mammography may not be recommended at the discretion of the treating physician. Guidelines are necessary to standardize cancer care but require additional adaption to tailor them to the clinical context.

#### Patient-Facing Barriers

Approximately half of HCPs reported regularly modifying clinical recommendations based on patients’ ability to comply. Glaringly, less than 10% of the population in Nigeria has health insurance, and most breast cancer care is paid for out of pocket by the patient. As a result, 68% to 95% of affected households experience catastrophic health care expenditure.^26^ There is an urgent need, therefore, to make cancer care more affordable in Nigeria. Additional, well-documented patient-facing barriers include distance to health care facilities, lack of coordination between levels and sectors of the health care system, limited breast health awareness, and reliance on traditional healers.^27,28,29,30,31^ Some of these barriers could be addressed by culturally tailored, multilevel patient navigation programs.^32^ The HCPs in our study and Nigeria’s National Strategic Cancer Control Plan highlight patient navigation as a priority for improving guideline-adherent breast cancer care.^9^

### Future Research Directions

**Figure 2.**
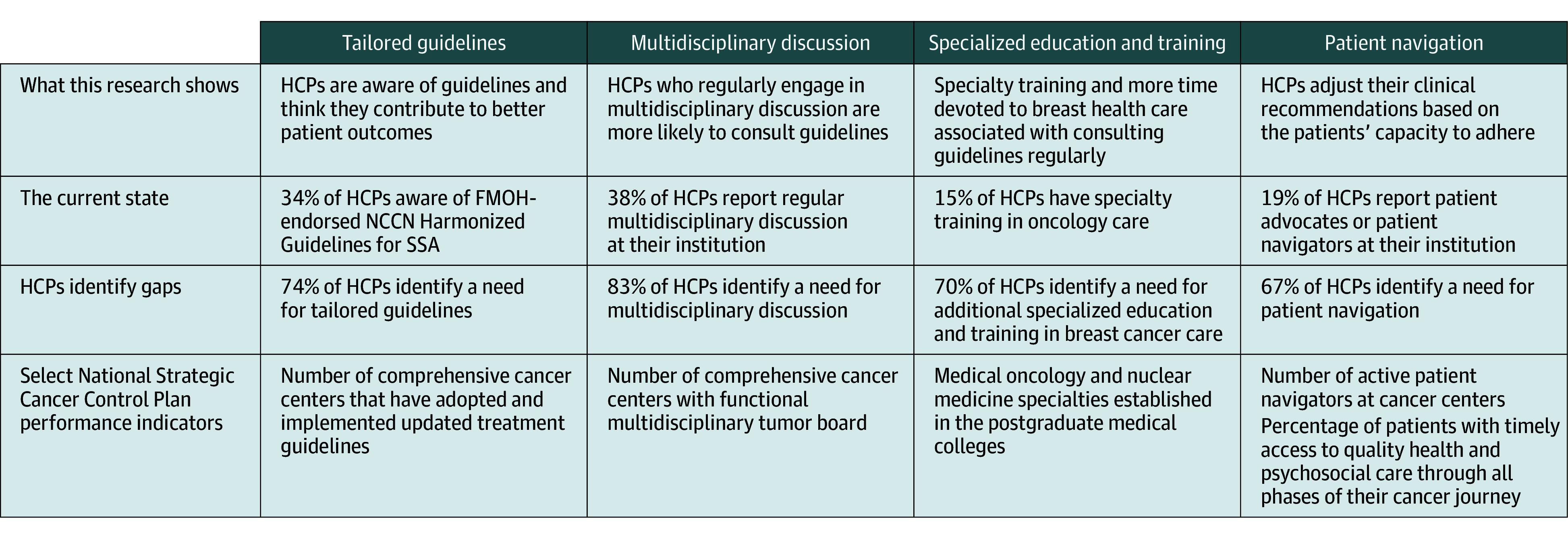
Health Care Practitioner (HCP) Priorities and the National Strategic Cancer Control Plan of Nigeria FMOH indicates Federal Ministry of Health; NCCN, National Comprehensive Cancer Network; SSA, Sub-Saharan Africa.

Strategies identified by HCPs, such as regular MDT discussions, more tailored guidelines (or increasing awareness of existing tailored guidelines), additional education or training in breast cancer care, and patient navigation programs, align with FMOH priorities set in the National Strategic Cancer Control Plan of Nigeria (Figure 2).^9^ In the next steps of this sequential mixed-methods research, focus groups with HCPs across the breast cancer care continuum will further examine factors influencing guideline use and preferences for intervention. CFIR constructs will guide the discussion, with particular attention to domains not specifically addressed in the HCP questionnaire (individuals involved and implementation process). Although most HCPs think that their organization is open to new initiatives, they also report a lack of institutional and structural support for implementation. Tailored, sustainable strategies are needed to address the identified barriers effectively.

### Limitations

This study has some limitations. We did not directly measure patient receipt of guideline-concordant care; an HCP reporting that a guideline was consulted does not necessarily ensure delivery of guideline-adherent care. Rather, this research represents a first step in understanding factors associated with guideline use, a prerequisite for the standardization of breast cancer care. The African Research Group for Oncology (ARGO) is prospectively evaluating receipt of guideline-adherent breast cancer care at a patient level across diverse institutions in Nigeria.

HCPs who are part of a national organization (including ARGO) may have been more likely to receive the questionnaire and respond to it, resulting in selection bias. HCPs may also be more likely to have access to MDT discussions, a patient database, and other resources through these organizations. Although we tried to capture a diverse cohort, most participants resided in urban areas, practiced at academic institutions, and engaged in research. This may limit the generalizability of our findings to other settings in Nigeria.

Because this questionnaire relied on self-reporting, HCPs may have overstated their awareness or use of guidelines. We attempted to mitigate these sources of bias by targeting HCPs across geopolitical zones and facilities and by questionnaire anonymization. Additionally, some of the associations estimated in our modeling may show high variance due to sparse data availability, reflected by wide CIs (eg, fellowship in cancer care).

## Conclusions

In this survey study of 277 HCPs across Nigeria, half reported routinely consulting guidelines, and only one-third were aware of the Harmonized Guidelines, which are resource adapted and endorsed by the FMOH. Approximately one-third of HCPs reported engaging in regular breast cancer MDT discussions; guideline awareness and consultation increased as frequency of MDT participation increased. Opportunities for disseminating knowledge, such as MDT discussions and fellowship training, are essential to standardize breast cancer care delivery in Nigeria. Access to regular MDT discussions has the potential to facilitate knowledge exchange across HCP types and improve awareness and use of resource-adapted guidelines.

## Notes

Supplement 1. **eAppendix.** Questionnaire **eTable 1.** Participation in Multidisciplinary Discussion **eTable 2.** Workflow Process **eFigure.** Forest Plot of Multivariable Associations With Consulting Guidelines Regularly

Supplement 2.
Data Sharing Statement
