# Supplementary material for: Health Care Professional Adherence to Breast Cancer Management Guidelines in Nigeria
Source: JAMA Netw Open. 2025 Feb 12;8(2):e2459614. doi: 10.1001/jamanetworkopen.2024.59614 (PMC11822546; doi:10.1001/jamanetworkopen.2024.59614)
Supplement: Supplement 1. — eAppendix. Questionnaire eTable 1. Participation in Multidisciplinary Discussion eTable 2. Workflow Process eFigure. Forest Plot of Multivariable Associations With Consulting Guidelines Regularly [file jamanetwopen-e2459614-s001.pdf]

Supplemental Online Content

Romanoff A, Olasehinde O, Lynch K, et al. Health care practitioner adherence to breast cancer management guidelines in Nigeria. *JAMA Netw Open*. Published online February 12, 2025. doi:10.1001/jamanetworkopen.2024.59614

eAppendix. Questionnaire

eTable 1. Participation in Multidisciplinary Discussion

eTable 2. Workflow Process

eFigure. Forest Plot of Multivariable Associations With Consulting Guidelines Regularly

This supplemental material has been provided by the authors to give readers additional information about their work.

© 2025 Romanoff A et al. *JAMA Network Open*.

eAppendix. Questionnaire

Questionnaire development

Utilization of BC guidelines in patient care delivery by a national sample of Nigerian health care providers (HCPs) was evaluated by a questionnaire developed by a multinational, multidisciplinary team, guided by the well-established Consolidated Framework for Implementation Research (CFIR).[11] Led by a breast surgical oncologist in the U.S. (AR) and a general surgeon in Nigeria (OO), the questionnaire development team included a radiologist (AO), pathologists (BO, OO) and a clinical oncologist (SF) practicing in Nigeria, surgical oncologists with global cancer leadership expertise (OA, PK, BA), a health services researcher with expertise in large-scale data collection in SSA (RV), as well as a qualitative methodologist with expertise in questionnaire development (KL), and a behavioral scientist with expertise in implementation science in global cancer care (JO).

The questionnaire consists of 4 sections: provider characteristics, institution characteristics, perceived barriers and facilitators to guideline adherence, and practice patterns based on relevant clinical scenarios. Demographic and practice information were collected. The guideline adherence section was informed by the Consolidated Framework for Implementation Research (CFIR), which assesses determinants of implementation across 5 domains (the intervention, inner setting [practice], outer setting [system], individuals involved, and implementation process), emphasizing the organizational context. CFIR has been employed to improve understanding of factors associated with healthcare delivery and can be modified for use in low- and middle-income countries (LMICs).[12-14] The questionnaire draws from three of the CFIR

© 2025 Romanoff A et al. *JAMA Network Open*.

domains (intervention, inner setting and outer setting) to provide structure for understanding barriers and facilitators of guideline adherence and measuring variation across providers. Clinical practice scenarios were tailored for the four clinical specialties (surgeons, clinical oncologists, radiologists, pathologists) and are reported separately.

The questionnaire was pilot tested among 8 HCPs in Nigeria (2 from each specialty). The qualitative methodologist (KL) engaged each respondent in 30–60-minute cognitive debriefing interviews to assess questionnaire comprehension and content validity and to elicit specialty-specific feedback. The final questionnaire was modified based on provider input, including reduction of overall items and tailoring of clinical scenarios. Pilot questionnaire results were excluded from the final analysis.

© 2025 Romanoff A et al. *JAMA Network Open*.

Breast Cancer Clinical Care in Nigeria
HEALTHCARE PROVIDER QUESTIONNAIRE

<u>REDCap Introduction</u>:

Thank you for your time and participation in this study examining current breast cancer clinical practices in Nigeria. The goal of this research is to develop contextually relevant interventions to improve breast cancer care in Nigeria. This survey will take approximately 15 minutes to complete.

The responses you provide to this survey will be anonymous. Each survey response will be assigned a unique code and any information that identifies you will be removed prior to data analysis. Responses to this survey will only be reported in aggregate.

If you have any questions, please contact the study Principal Investigators.

Do you agree to participate in this survey?

- o Yes [CONTINUE TO SURVEY]
- o No [END SURVEY]

PROVIDER

1) Provider age: ______

2) Gender:
   a) Male
   b) Female

3) Profession:
   a) Surgeon
   b) Medical/clinical/radiation oncologist
   c) Pathologist
   d) Radiologist

4) Professional status:
   a) Consultant
   b) Senior registrar
   c) Junior registrar

5) Number of years in practice (not including training/residency): _____ years

6) Have you completed a fellowship in oncology?
   a) No
   b) Yes
      1. How long was the fellowship?
         a. <3 months
         b. 3-12 months
         c. >12 months

© 2025 Romanoff A et al. *JAMA Network Open*.

7) Estimated percentage of your total practice time specifically devoted to breast health care: _______%

8) How often do you routinely engage in <u>multi-disciplinary</u> discussion regarding breast cancer patients?
   a) Weekly
   b) Biweekly
   c) Monthly
   d) Case-by-case basis
   e) None

9) Were you actively engaged in research within the last 5 years:
   a) No
   b) Some (accrues patients to studies, co-investigator and/or middle author on published manuscripts)
   c) A lot (leads studies, principal investigator and/or first/last author on published manuscripts)

<u>INSTITUTION</u>

10) Facility type:
   a) Public university teaching hospital
   b) Private university teaching hospital
   c) Federal medical center
   d) State specialist hospital
   e) General hospital
   f) Private specialist hospital
   g) Other: [Free Text]__________________________

11) Geopolitical zone:
   a) South-east
   b) South-south
   c) South-west
   d) North-central
   e) North-east
   f) North-west

12) How would you describe your facility location?
   a) City
   b) Town/semi-dense area
   c) Rural

13) Approximately how many inpatient beds are in your hospital? ___________ beds

14) How far is your facility located from the next referral center (driving distance)?
   a) <1 hour
   b) 1-3 hours
   c) >3 hours
   d) I don't know

© 2025 Romanoff A et al. *JAMA Network Open*.

15) Which of the following healthcare professionals provide breast cancer care at your institution (select all that apply)?
   a) Surgeon
   b) Pathologist
   c) Pathology laboratory technician
   d) Radiologist
   e) Radiology technologist
   f) Medical, clinical and/or radiation oncologist
   g) Palliative care
   h) Nurse
   i) Pharmacist
   j) Psychiatrist/psychologist/mental health professional
   k) Social worker
   l) Patient navigator or patient advocate (someone who supports patients and helps *guide them through the health care system)*
   m) Physical therapist
   n) Nutritionist/dietician
   o) Other: [Free Text]__________________________

GUIDELINES

16) Are you aware of any established institutional, national or international guidelines for breast cancer care?
   a) Yes
   b) No

17) *[If yes to 16]* Which ones (select all that apply)?
   a) American Society of Clinical Oncology (ASCO)
   b) American Society for Radiation Oncology (ASTRO)
   c) Breast Health Global Initiative (BHGI)
   d) Cancer Care Ontario (CCO)
   e) European Society of Medical Oncology (ESMO)
   f) National Comprehensive Cancer Center (NCCN) guidelines
   g) NCCN Resource-stratified guidelines
   h) NCCN Harmonized Guidelines for sub-Saharan Africa
   i) Saint Gallen International Consensus Conference
   j) Tailored institutional guidelines
   k) Other: [Free Text]__________________________

18) *[If yes to 16]* How often do you consult breast cancer guidelines in your clinical practice?
   a) Never
   b) Rarely
   c) Sometimes
   d) Most of the time
   e) Always

19) *[If "b," "c," "d," or "e" to 18]* How do you access these guidelines (select all that apply)?
   a) Online

© 2025 Romanoff A et al. *JAMA Network Open*.

b) Paper copy
c) Mobile application (app)
d) Other: [Free Text]___________________________

Breast cancer guidelines *are recommendations intended to optimize patient care. Please indicate the extent to which you* *agree or disagree* *with the following statements:*

20) I believe breast cancer guidelines are applicable to the patient care setting in which I practice.

1	2	3	4	5
Strongly disagree	Disagree	Neither agree nor disagree	Agree	Strongly agree

21) I think breast cancer guidelines contribute to better patient outcomes.

1	2	3	4	5
Strongly disagree	Disagree	Neither agree nor disagree	Agree	Strongly agree

22) I think breast cancer guidelines are too complex to be useful for helping me make choices about patient care.

1	2	3	4	5
Strongly disagree	Disagree	Neither agree nor disagree	Agree	Strongly agree

23) In my current practice, I have sufficient resources to provide care adherent to breast cancer guidelines.

1	2	3	4	5
Strongly disagree	Disagree	Neither agree nor disagree	Agree	Strongly agree

24) To ensure the care I am providing is adherent to breast cancer guidelines, I would need these additional tools/resources (select all that apply)?
a) Physically accessible guidelines
b) Guidelines tailored to my practice setting
c) Additional specialized education/training in breast cancer care
d) Regular structured multi-disciplinary discussions (MDT) about patient care
e) Infrastructure for communication between providers (consultation or bidirectional feedback)
f) Infrastructure for patient referrals/navigation through the healthcare system
g) Other: [Free Text]___________________________

The following questions ask about your ***work-flow processes:***

25) Do you receive any feedback about the care you provide (select all that apply)?
a) Formal from department/institution
b) Informal from supervisor/colleagues
c) Written report
d) Targeted metrics/data-driven feedback
e) Direct patient comments

© 2025 Romanoff A et al. *JAMA Network Open*.

f) None/ I don't receive feedback
g) Other: [Free Text]___________________________

26) Do you have an institutional database or registry to collect information on breast cancer patients at your center?
a) Yes
b) No
c) I don't know

27) How often do you have access to an electronic data system that allows you to track patients? (e.g., electronic medical record)

1	2	3	4	5
Never	Rarely	Sometimes	Most of the time	Always

28) To what extent do you feel like you can try new things to improve your work processes?

1	2	3	4	5
Never	Rarely	Sometimes	Most of the time	Always

29) How do you typically find out about practice-changing innovations (learnings that would prompt you to change your practice and/or scientific discovery within your field of practice) (select all that apply)?
a) Institutional meetings
b) National conferences (e.g., African Research Group for Oncology, ARGO)
c) International conferences (e.g., African Organisation for Research and Training in Cancer, AORTIC)
d) Professional trainings
e) Personal reading of the medical literature
f) Interaction with colleagues
g) Other: [Free Text]___________________________

30) Generally, to what extent does your organization support you in trying new things or adopt new innovations to improve your work processes?

1	2	3	4	5
Never	Rarely	Sometimes	Most of the time	Always

31) What do you think would be the level of receptivity by your organization's leadership (hospital administrators/department chair) to implementing structured breast cancer care programs to promote guideline adherence?

1	2	3	4	5
Not at all receptive	Slightly receptive	Somewhat receptive	Moderately receptive	Extremely receptive

© 2025 Romanoff A et al. *JAMA Network Open*.

32) To what extent do you network with colleagues or people in similar professions/positions outside your clinical practice setting?

1	2	3	4	5
Never	Rarely	Sometimes	Most of the time	Always

33) How do you connect with colleagues to discuss complex clinical cases (select all that apply)?
   a) Departmental meetings
   b) Formal multidisciplinary meetings (MDT)/institutional tumor board conferences
   c) Virtual tumor board conference (e.g., Project ECHO)
   d) National/international conferences
   e) Professional society online fora/listservs
   f) WhatsApp or other messaging platform
   g) Social media (Facebook, Instagram, Twitter, etc.)
   h) Informal discussion with colleagues
   i) Other: [Free Text]__________________________
   j) None of the above

© 2025 Romanoff A et al. *JAMA Network Open*.

CLINICAL PRACTICE- SURGEON

1. Approximately how many breast cancer surgeries (for treatment) do you (personally) perform per year?
   ____ surgeries

2. How many general surgeons are in your facility?
   a) *0*
   b) *1-2*
   c) *3-5*
   d) *>5*

3. How many specialty-trained breast surgeons are in your facility?
   a) *0*
   b) *1-2*
   c) *3-5*
   d) *>5*

Clinical cases:

4. Generally, in your practice, how often do you adjust your clinical recommendations based on whether the patient will be able to follow them?

1	2	3	4	5
Never	Rarely	Sometimes	Most of the time	Always

A 42-year-old healthy female presents with a right breast complaint. Examination reveals a 5cm breast mass with mobile axillary lymphadenopathy…

5. What would you recommend as the first step for this patient?
   a) Breast imaging
   b) Needle biopsy
   c) Excision
   d) Mastectomy
   e) Refer patient to another facility
   f) Watchful waiting

6. We want to understand what happens to patients in your system- What investigation(s) is this patient most likely to receive (choose the response(s) that best fit your **current practice**, select all that apply)?
   a) Mammogram
   b) Breast ultrasound
   c) Axillary ultrasound
   d) Breast MRI
   e) Fine needle aspiration breast biopsy (FNA)
   f) Tru-cut/core breast biopsy
   g) Incisional breast biopsy
   h) Excisional breast biopsy
   i) Axillary lymph node needle biopsy

© 2025 Romanoff A et al. *JAMA Network Open*.

7. How often are the following test(s) available in your setting (select the responses that best fit your **current practice)**?

	Never	Rarely	Sometimes	Most of the time	Always
Mammogram					
Breast ultrasound					
Axillary ultrasound					
Breast MRI					
Chest x-ray					
Abdominal ultrasound					
CT chest/abdomen/pelvis					
Bone scan					
PET scan					
Fine needle aspiration biopsy (FNA)					
Tru-cut/core biopsy					
Ultrasound-guided biopsy					
Stereotactic (mammography-guided) biopsy					
Clip/marker placement during breast biopsy					
Axillary lymph node needle biopsy					
Clip/marker placement during axillary lymph node biopsy					

8. How often are the following test(s) available in your setting (select the responses that best fit your **current practice)**?

	Never	Rarely	Sometimes	Most of the time	Always
Histopathology					
Cytology					
Estrogen receptor (ER) and progesterone receptor (PR) status					
Her2 receptor status					
Genomic testing of the tumor (e.g., Oncotype DX, MammaPrint)					
Genetic testing of the patient (e.g., BRCA)					

9. How often do you have a pathologic diagnosis of cancer before beginning treatment (systemic medication or definitive surgery) (select the response that best fits your **current practice)**?

1	2	3	4	5
Never	Rarely	Sometimes	Most of the time	Always

© 2025 Romanoff A et al. *JAMA Network Open*.

10. How often do you have immunohistochemistry (ER/PR) results before beginning treatment (systemic medication or definitive surgery) (select the response that best fits your **current practice)**?

1	2	3	4	5
Never	Rarely	Sometimes	Most of the time	Always

Based on the initial work-up, the 42-year-old patient above is found to have invasive ductal carcinoma (5cm) that is triple negative (ER-/PR-/Her2-) with a positive axillary lymph node…

11. Which of the following additional investigations would you recommend at this time (choose the response(s) that best fit your **current practice**, select all that apply)?
    a) Baseline blood investigations
    b) Pregnancy test
    c) Chest x-ray
    d) Abdominal ultrasound
    e) CT chest/abdomen/pelvis
    f) Bone scan
    g) PET scan
    h) Cranial CT
    i) None

The patient above does not have any evidence of distant metastatic disease…

12. Which of the following modalities would you recommend as the <u>first</u> intervention for this patient?
    a) Surgery
    b) Hormonal therapy
    c) Systemic chemotherapy
    d) Radiation
    e) Watchful waiting

13. How often are the following procedure(s) performed in your setting (select the responses that best fit your **current practice)**?

	Never	Rarely	Sometimes	Most of the time	Always
Needle/wire localization					
Breast conserving surgery					
Sentinel lymph node biopsy with blue dye					
Sentinel lymph node biopsy with radioactive isotope					
Autologous breast reconstruction					
Implant-based breast reconstruction					

© 2025 Romanoff A et al. *JAMA Network Open*.

14. What axillary surgery do you usually recommend for patients with positive lymph nodes (cN1)?
    a) Surveillance
    b) Excise abnormal-appearing lymph nodes
    c) Sentinel lymph node biopsy (using mapping with blue dye or radioisotope)
    d) Complete axillary lymph node dissection
    e) I don't usually recommend axillary surgery for patients with positive lymph nodes

15. In your practice, when you receive the report of your axillary lymph node dissection specimen from pathology, what do you consider to be an adequate number of lymph nodes removed?
    ______ nodes

16. How often are the following medications available in your setting (select the responses that best fit your **current practice)**?

	Never	Rarely	Sometimes	Most of the time	Always	Don't Know
Capecitabine						
Carboplatin						
Cyclophosphamide						
Docetaxel						
Doxorubicin						
Epirubicin						
Fluorouracil						
Methotrexate						
Paclitaxel						
Vinorelbine						
Trastuzumab						
Anastrozole (or letrozole)						
Leuprorelin (or goserelin)						
Tamoxifen						
CDK4/6 inhibitor						
Anti-emetic (e.g. ondansetron, metoclopramide, aprepitant)						
H2 receptor antagonist						
Steroid (e.g. dexamethasone)						
Fentanyl patch						
Methadone						
Morphine						

17. Who administers chemotherapy in your institution (select all that apply)?
    a) Nurses
    b) Medical officers
    c) Medical oncologists
    d) Clinical/radiation oncologists

© 2025 Romanoff A et al. *JAMA Network Open*.

e) Surgeons
f) Residents/registrars

18. How often do patients who are prescribed chemotherapy complete it?

1	2	3	4	5
Never	Rarely	Sometimes	Most of the time	Always

19. How often do patients complete recommended breast cancer surgery?

1	2	3	4	5
Never	Rarely	Sometimes	Most of the time	Always

20. To what extent do you agree/disagree with the following statement: "If a patient has a complete clinical response to neoadjuvant chemotherapy (NAC), they do not require surgery."

1	2	3	4	5
Strongly Disagree	Disagree	Neither agree nor disagree	Agree	Strongly Agree

A patient with de novo metastatic breast cancer (T2N1M1 with liver metastases) comes to see you:

21. We want to understand what happens to patients in your system- What treatment <u>would you recommend</u> for this patient?
    a) Surgery to remove the breast mass and/or axillary lymph nodes
    b) Systemic therapy
    c) Radiotherapy
    d) Watchful waiting

22. In your practice, in a patient with known metastatic breast cancer, how often do you perform surgical resection of the primary tumor?

1	2	3	4	5
Never	Rarely	Sometimes	Most of the time	Always

23. To what extent do you agree/disagree with the following statement: In a patient with metastatic disease, surgery to remove the primary tumor improves survival.

1	2	3	4	5
Strongly disagree	Disagree	Neither agree nor disagree	Agree	Strongly agree

24. How often do patients receive radiation when it is recommended?

1	2	3	4	5
Never	Rarely	Sometimes	Most of the time	Always

25. Is there a radiotherapy machine in your institution?

© 2025 Romanoff A et al. *JAMA Network Open*.

a) Yes
   1. If yes, how often does this radiotherapy machine function?

1	2	3	4	5
Never	Rarely	Sometimes	Most of the time	Always

b) No
   1. If no, approximately how far (in hours of ground travel) is the closest functioning radiation facility to your institution? ____ hours

26. In your practice, how often do breast cancer patients return for a follow-up examination in the first year following completion of treatment?

1	2	3	4	5
Never	Rarely	Sometimes	Most of the time	Always

27. In your practice, how often do breast cancer patients return for an examination at least once per year for the first five years following completion of treatment?

1	2	3	4	5
Never	Rarely	Sometimes	Most of the time	Always

© 2025 Romanoff A et al. *JAMA Network Open*.

<u>**CLINICAL PRACTICE- MEDICAL/CLINICAL/RADIATION ONCOLOGIST**</u>

1) Approximately how many breast cancer patients do you (personally) prescribe chemotherapy per year?
   ____ patients

2) Approximately how many breast cancer patients do you (personally) treat with radiotherapy per year?
   ____ patients

3) How many general medical/clinical/radiation oncologists are in your facility?
   a) 0
   b) 1-2
   c) 3-5
   d) >5

4) How many specialty trained breast medical/clinical/radiation oncologists are in your facility?
   a) 0
   b) 1-2
   c) 3-5
   d) >5

Clinical cases:

5) Generally, in your practice, how often do you adjust your clinical recommendations based on whether the patient will be able to follow them?

1	2	3	4	5
Never	Rarely	Sometimes	Most of the time	Always

A 42-year-old healthy female presents with a right breast complaint. Examination reveals a 5cm breast mass with mobile axillary lymphadenopathy…

6) What <u>would you recommend</u> as the first step for this patient?
   a) Breast imaging
   b) Needle biopsy
   c) Refer patient to surgeon
   d) Refer patient to another facility
   e) Watchful waiting

7) We want to understand what happens to patients in your system. What additional investigations is this patient <u>most likely to receive</u>? (Choose the response(s) that best fit your **current practice**, select all that apply).
   a) Mammogram
   b) Breast ultrasound
   c) Axillary ultrasound
   d) Breast MRI
   e) Fine needle aspiration breast biopsy (FNA)
   f) Tru-cut/core breast biopsy

© 2025 Romanoff A et al. *JAMA Network Open*.

g) Incisional breast biopsy
h) Excisional breast biopsy
i) Axillary lymph node needle biopsy

8) How often are the following test(s) available in your setting (select the responses that best fit your **current practice)**?

	Never	Rarely	Sometimes	Most of the time	Always
Mammogram					
Breast ultrasound					
Axillary ultrasound					
Breast MRI					
Chest x-ray					
Abdominal ultrasound					
CT chest/abdomen/pelvis					
Bone scan					
PET scan					
Fine needle aspiration biopsy (FNA)					
Tru-cut/core biopsy					
Ultrasound-guided biopsy					
Stereotactic (mammography-guided) biopsy					
Clip/marker placement during breast biopsy					
Axillary lymph node needle biopsy					
Clip/marker placement during axillary lymph node biopsy					

9) How often are the following test(s) available in your setting (select the responses that best fit your **current practice)**?

	Never	Rarely	Sometimes	Most of the time	Always
Histopathology					
Cytology					
Estrogen receptor (ER) and progesterone receptor (PR) status					
Her2 receptor status					

© 2025 Romanoff A et al. *JAMA Network Open*.

Genomic testing of the tumor (e.g. Oncotype DX, MammaPrint)					
Genetic testing of the patient (e.g. BRCA)					

10) How often do you have a pathologic diagnosis of cancer before beginning treatment (systemic medication or definitive surgery) (select the response that best fits your **current practice)**?

1	2	3	4	5
Never	Rarely	Sometimes	Most of the time	Always

11) How often do you have immunohistochemistry (ER/PR) results before beginning treatment (systemic medication or definitive surgery) (select the response that best fits your **current practice)**?

1	2	3	4	5
Never	Rarely	Sometimes	Most of the time	Always

Based on the initial work-up, the 42-year-old patient above is found to have invasive ductal carcinoma (5cm) that is triple negative (ER-/PR-/Her2-) with a positive axillary lymph node…

12) Which of the following additional investigations would you recommend at this time (choose the response(s) that best fit your **current practice**, select all that apply)?
    a) Baseline blood investigations
    b) Pregnancy test
    c) Chest x-ray
    d) Abdominal ultrasound
    e) CT chest/abdomen/pelvis
    f) Bone scan
    g) PET scan
    h) Cranial CT
    i) None

The patient above does not have any evidence of distant metastatic disease…

13) Which of the following modalities would you recommend as the <u>first</u> intervention for this patient?
    a) Surgery
    b) Hormonal therapy
    c) Systemic chemotherapy
    d) Radiation
    e) Watchful waiting

14) How often are the following medications available in your setting (select the responses that best fit your **current practice)**?

	Never	Rarely	Sometimes	Most of the time	Always	Don't Know
Capecitabine						

© 2025 Romanoff A et al. *JAMA Network Open*.

Carboplatin						
Cyclophosphamide						
Docetaxel						
Doxorubicin						
Epirubicin						
Fluorouracil						
Methotrexate						
Paclitaxel						
Vinorelbine						
Trastuzumab						
Anastrozole (or letrozole)						
Leuprorelin (or goserelin)						
Tamoxifen						
CDK4/6 inhibitor						
Anti-emetic (e.g. ondansetron, metoclopramide, aprepitant)						
H2 receptor antagonist						
Steroid (e.g. dexamethasone)						
Fentanyl patch						
Methadone						
Morphine						

15) Who administers chemotherapy in your institution (select all that apply)?
   a) Nurses
   b) Medical officers
   c) Medical oncologists
   d) Clinical/radiation oncologists
   e) Surgeons
   f) Residents/registrars

16) How often do patients who are prescribed chemotherapy complete it?

1	2	3	4	5
Never	Rarely	Sometimes	Most of the time	Always

17) To what extent do you agree/disagree with the following statement: “If a patient has a complete clinical response to neoadjuvant chemotherapy (NAC), they do not require surgery.”

1	2	3	4	5
Strongly Disagree	Disagree	Neither agree nor disagree	Agree	Strongly Agree

A 60-year-old treatment naïve patient with metastatic breast cancer (T2N1M1) with liver metastases comes to see you. Immunohistochemistry results show that her cancer is hormone receptor positive.

© 2025 Romanoff A et al. *JAMA Network Open*.

18) We want to understand what happens to patients in your system- What treatment <u>would you recommend</u> for this patient?
   a) Surgery to remove the breast mass and/or axillary lymph nodes
   b) Systemic therapy
   c) Radiotherapy
   d) Watchful waiting

19) Regarding the patient above, to what extent do you agree/disagree with the following statements?

   a) Combination agent chemotherapy is generally preferred to single agent.

1	2	3	4	5
Strongly disagree	Disagree	Neither agree nor disagree	Agree	Strongly agree

   b) This patient would benefit from hormonal therapy.

1	2	3	4	5
Strongly disagree	Disagree	Neither agree nor disagree	Agree	Strongly agree

   c) Systemic treatment can prolong survival, improve quality of life, and also cure the disease.

1	2	3	4	5
Strongly disagree	Disagree	Neither agree nor disagree	Agree	Strongly agree

   d) There is the need to be aggressive with systemic therapy, to increase the chance of cure.

1	2	3	4	5
Strongly disagree	Disagree	Neither agree nor disagree	Agree	Strongly agree

   e) In a patient with metastatic disease, surgery to remove the primary tumor improves survival.

1	2	3	4	5
Strongly disagree	Disagree	Neither agree nor disagree	Agree	Strongly agree

20) How often do patients receive radiation when it is recommended?

1	2	3	4	5
Never	Rarely	Sometimes	Most of the time	Always

21) Is there a radiotherapy machine in your institution?
   a) Yes
      1. If yes, how often does this radiotherapy machine function?

1	2	3	4	5
Never	Rarely	Sometimes	Most of the time	Always

© 2025 Romanoff A et al. *JAMA Network Open*.

b) No
   1. If no, approximately how far (in hours of ground travel) is the closest functioning radiation facility to your institution? ____ hours

22) In your practice, how often do breast cancer patients return for a follow-up examination in the first year following completion of treatment?

1	2	3	4	5
Never	Rarely	Sometimes	Most of the time	Always

23) In your practice, how often do breast cancer patients return for an examination at least once per year for the first five years following completion of treatment?

1	2	3	4	5
Never	Rarely	Sometimes	Most of the time	Always

© 2025 Romanoff A et al. *JAMA Network Open*.

CLINICAL PRACTICE- RADIOLOGIST

1) Approximately how many breast imaging studies do you (personally) read per year?
   a) Mammogram _____
   b) Breast ultrasound ______
   c) Breast MRI _____

2) How many radiologists are in your facility?
   a) 0
   b) 1-2
   c) 3-5
   d) >5

3) Approximately how many breast imaging studies are performed per year at your institution?
   a) Mammogram _____
   b) Breast ultrasound ______
   c) Breast MRI _____

Clinical cases:

4) Generally, in your practice, how often do you adjust your recommendations based on whether the patient will be able to follow them?

1	2	3	4	5	6
Never	Rarely	Sometimes	Most of the time	Always	Not applicable to my specialty

A 42-year-old healthy female presents with a right breast complaint. Examination reveals a 5cm breast mass with mobile axillary lymphadenopathy...

5) We want to understand what happens to patients in your system- what imaging studies would you recommend as the first step for this patient (choose the response(s) that best fit your **current practice**, select all that apply)?
   a) Mammogram
   b) Breast ultrasound
   c) Axillary ultrasound
   d) Breast MRI
   e) Chest x-ray
   f) Abdominal ultrasound
   g) CT chest/abdomen/pelvis
   h) Bone scan
   i) PET scan
   j) Cranial CT
   k) None

6) How often are the following test(s) available in your setting (select the responses that best fit your **current practice)**?

© 2025 Romanoff A et al. *JAMA Network Open*.

	Never	Rarely	Sometimes	Most of the time	Always
Mammogram					
Breast ultrasound					
Axillary ultrasound					
Breast MRI					
Chest x-ray					
Abdominal ultrasound					
CT chest/abdomen/pelvis					
Bone scan					
PET scan					
Fine needle aspiration biopsy (FNA)					
Tru-cut/core biopsy					
Ultrasound-guided biopsy					
Stereotactic (mammography-guided) biopsy					
Clip/marker placement during breast biopsy					
Axillary lymph node needle biopsy					
Clip/marker placement during axillary lymph node biopsy					

7) How often do you include the BIRADS (Breast Imaging Reporting and Data System) category in your mammogram or breast ultrasound report (select the response that best fits your **current practice**)?

1	2	3	4	5
Never	Rarely	Sometimes	Most of the time	Always

The patient above undergoes an ultrasound, which reveals an irregular 5cm mass with intense posterior acoustic shadowing in the upper and lower inner quadrants of the right breast and rounded lymph nodes with loss of their fatty hilum in the ipsilateral axilla.

8) What is the BIRADS category for this patient?
   a) *0*
   b) *1*
   c) *2*
   d) *3*
   e) *4*
   f) *5*

© 2025 Romanoff A et al. *JAMA Network Open*.

g) *6*

9) What would you recommend as a next step for this patient?
   a) Watchful waiting
   b) Repeat/additional imaging now
   c) Short-term follow-up (6 months)
   d) Needle biopsy
   e) Excision by surgeon

10) What would you recommend for the axillary lymph nodes in this patient?
   a) Watchful waiting
   b) Repeat/additional imaging now
   c) Short-term follow-up (6 months)
   d) Needle biopsy
   e) Excision by surgeon

11) How often are clip markers placed in breast lesions after biopsy in your institution (select the responses that best fit your **current practice)**?

1	2	3	4	5
Never	Rarely	Sometimes	Most of the time	Always

12) How often are clip markers placed in axillary lymph nodes after biopsy in your institution (select the responses that best fit your **current practice)**?

1	2	3	4	5
Never	Rarely	Sometimes	Most of the time	Always

The biopsy result for the patient above returns invasive ductal carcinoma and she is planned to receive neo-adjuvant chemotherapy.

13) Which of the following additional imaging modalities would you recommend at this time (choose the response(s) that best fit your **current practice**, select all that apply)?
   a) Chest x-ray
   b) Abdominal ultrasound
   c) CT chest/abdomen/pelvis
   d) Bone scan
   e) PET scan
   f) Cranial CT
   g) None

14) Are images and reports archived electronically in your institution (select the responses that best fit your **current practice)**?

1	2	3	4	5
Never	Rarely	Sometimes	Most of the time	Always

© 2025 Romanoff A et al. *JAMA Network Open*.

15) In your practice, what do you do when there is discordance/disagreement between radiology and pathology findings?
   a) Watchful waiting
   b) Re-biopsy
   c) Recommend excisional biopsy
   d) Decision is made by referring healthcare provider
   e) Decision is made at a formal multidisciplinary (MDT) meeting

16) How would you manage microcalcifications detected on screening mammography?
   b) Watchful waiting
   c) Palpation-guided biopsy
   d) Image-guided biopsy
   e) Wire-localized excision
   f) Refer to another facility

18) In your practice, what is the estimated time from imaging recommendation/order to completion of the radiology report?
   ____ weeks

19) In your practice, what is the estimated time from biopsy recommendation/order to completion of the biopsy?
   ____ weeks

20) In your practice do patients receive reports directly?

1	2	3	4	5
Never	Rarely	Sometimes	Most of the time	Always

© 2025 Romanoff A et al. *JAMA Network Open*.

CLINICAL PRACTICE- PATHOLOGIST

1) Approximately how many total breast pathology cases (benign or malignant) do you (personally) read per year?
   ____ cases

2) Approximately how many breast carcinoma diagnoses do you (personally) make per year?
   ____ diagnoses

3) How many general pathologists are in your facility?
   a) 0
   b) 1-2
   c) 3-5
   d) >5

4) How many specialty-trained breast pathologists are in your facility?
   a) 0
   b) 1-2
   c) 3-5
   d) >5

Clinical cases:

5) Generally, in your practice, how often do you adjust your recommendations based on whether the patient will be able to follow them?

1	2	3	4	5	6
Never	Rarely	Sometimes	Most of the time	Always	Not applicable to my specialty

A 42-year-old healthy female presents with a right breast complaint. Examination reveals a 5cm breast mass with mobile axillary lymphadenopathy. A trucut/core biopsy was taken from the breast and fixed in 10% neutral buffered formalin.

6) How often do you include the following in your **biopsy pathology report** (select the responses that best fit your **current practice)**?

	Never	Rarely	Sometimes	Most of the time	Always	Upon Request
Histology type						
Histology grade						
Presence/extent of ductal carcinoma in situ (DCIS)						
Presence of lymphovascular invasion (LVI)						
Ki67						

© 2025 Romanoff A et al. *JAMA Network Open*.

Estrogen receptor (ER) and progesterone receptor (PR) status						
Her2 receptor status						

The patient above is diagnosed with invasive ductal carcinoma, receives neoadjuvant chemotherapy and undergoes a modified radical mastectomy.

7) How often do you include the following in your **surgical pathology report of the breast** (select the responses that best fit your **current practice)**?

	Never	Rarely	Sometimes	Most of the time	Always	Upon Request
Histology type						
Histology grade						
Size of invasive cancer						
Presence/extent of ductal carcinoma in situ (DCIS)						
Presence of lymphovascular invasion (LVI)						
Ki67						
Treatment effect						
Margin status (positive/negative)						
Specific distance to margin						
Estrogen receptor (ER) and progesterone receptor (PR) status						
Her2 receptor status						
Pathological stage (pTNM)						

8) How often do you include the following in your **surgical pathology report of the axilla** (select the responses that best fit your **current practice)**?

	Never	Rarely	Sometimes	Most of the time	Always	Upon Request
Total number of lymph nodes						
Metastatic carcinoma present or absent						
Total number of positive/cancerous lymph nodes						

© 2025 Romanoff A et al. *JAMA Network Open*.

Presence of extracapsular extension						
Treatment effect						

9) How often is cytology available in your setting?

1	2	3	4	5
Never	Rarely	Sometimes	Most of the time	Always

10) How often is immunohistochemistry (IHC) available in your setting?

1	2	3	4	5
Never	Rarely	Sometimes	Most of the time	Always

11) When IHC is not available, what is often responsible (select all that apply)?
   a) Lack of reagents
   b) No trained personnel to perform
   c) Patient unable to afford cost
   d) Lack of electricity
   e) Other: [Free Text]___________________________

12) When IHC is not available, what do you do next?
   a) Send the tissue block to an outside laboratory
   b) Refer the patient for biopsy/testing elsewhere
   c) Do not report on IHC

13) In your setting, how often do you request/perform the following (select the responses that best fit your **current practice)**?
   a) I request ER/PR testing to be performed on breast cancer cases

1	2	3	4	5
Never	Rarely	Sometimes	Most of the time	Always

   b) I request Her2 testing to be performed on breast cancer cases

1	2	3	4	5
Never	Rarely	Sometimes	Most of the time	Always

   c) I correlate histological grade and hormone receptor status

1	2	3	4	5
Never	Rarely	Sometimes	Most of the time	Always

   d) I comment on internal/external controls

1	2	3	4	5
Never	Rarely	Sometimes	Most of the time	Always

© 2025 Romanoff A et al. *JAMA Network Open*.

e) I report % of staining of ER/PR positivity

1	2	3	4	5
Never	Rarely	Sometimes	Most of the time	Always

f) I comment on ER low positive cases (1-10%)

1	2	3	4	5
Never	Rarely	Sometimes	Most of the time	Always

g) FISH is performed for equivocal HER2 cases

1	2	3	4	5
Never	Rarely	Sometimes	Most of the time	Always

14) In your setting, how often does the surgeon/radiologist specify the location (quadrant/o'clock) of the breast biopsy?

1	2	3	4	5
Never	Rarely	Sometimes	Most of the time	Always

15) In your practice, how often do you view the radiology report?

1	2	3	4	5
Never	Rarely	Sometimes	Most of the time	Always

16) In your practice, how often do you ink your margins?

1	2	3	4	5
Never	Rarely	Sometimes	Most of the time	Always

17) In your practice, how often do you comment on radiology-pathology concordant or discordant cases?

1	2	3	4	5
Never	Rarely	Sometimes	Most of the time	Always

18) In your practice, what is the estimated time between receipt of a biopsy specimen and creation of final report?
_____ weeks

19) In your practice, what is the estimated time between receipt of a surgical specimen and creation of final report?
_____ weeks

© 2025 Romanoff A et al. *JAMA Network Open*.

20) In your practice do patients receive, or have access to, reports directly?

1	2	3	4	5
Never	Rarely	Sometimes	Most of the time	Always

© 2025 Romanoff A et al. *JAMA Network Open*.

IMPROVING BREAST CANCER CARE DELIVERY IN NIGERIA
SKIP LOGIC ENDS AND ALL PROVIDERS ANSWER THE REMAINING QUESTIONS:

1) What are the five most important factors that would improve **your ability** to care for patients with breast cancer (select **five**)?
   a) More healthcare providers to address time/workload constraints
   b) Regular educational update courses for healthcare providers
   c) Structured opportunities to discuss patient care with colleagues
   d) Patient support/navigation (to help *guide patients through the health care system)*
   e) Patient awareness/education
   f) An outpatient breast health clinic (for screening and early detection)
   g) A dedicated inpatient breast cancer care unit
   h) Clinical support staff
   i) An electronic medical record
   j) Institutional or national breast cancer patient data collection/registry
   k) Accessibility of imaging
   l) Accessibility of pathology (other than IHC)
   m) Accessibility of immunohistochemistry (IHC)
   n) Accessibility of medications
   o) Accessibility of surgery
   p) Accessibility of radiation
   q) Accessibility of palliative care services
   r) Accessibility of psycho-oncology services
   s) Programs to decrease the cost of care for patients

2) Of this list, what is the single most important factor that would improve **your ability** to care for patients with breast cancer (select **one**)?
   a) More healthcare providers to address time/workload constraints
   b) Regular educational update courses for healthcare providers
   c) Structured opportunities to discuss patient care with colleagues
   d) Patient support/navigation (to help *guide patients through the health care system)*
   e) Patient awareness/education
   f) An outpatient breast health clinic (for screening and early detection)
   g) A dedicated inpatient breast cancer care unit
   h) Clinical support staff
   i) An electronic medical record
   j) Institutional or national breast cancer patient data collection/registry
   k) Accessibility of imaging
   l) Accessibility of pathology (other than IHC)
   m) Accessibility of immunohistochemistry (IHC)
   n) Accessibility of medications
   o) Accessibility of surgery
   p) Accessibility of radiation
   q) Accessibility of palliative care services
   r) Accessibility of psycho-oncology services
   s) Programs to decrease the cost of care for patients

© 2025 Romanoff A et al. *JAMA Network Open*.

Is there any other information regarding your breast cancer care practices that would be important for us to know? [FREE TEXT]

Thank you for completing the survey. Are you interested in being contacted about a future focus group discussion to share more about your experience taking care of breast cancer patients in Nigeria? Your contact details will not be shared outside the study team, and will be disaggregated from your survey responses during data analysis:

- Yes, I would like to be contacted and understand that my contact details will be used only for focus group sampling and recruitment.
    - Email: ________________
    - Phone/WhatsApp: _________________
- No, I do not wish to be contacted for a future focus group.

© 2025 Romanoff A et al. *JAMA Network Open*.

eTable 1. Participation in Multidisciplinary Discussion

	Healthcare Providers<br>N=277
Engage in routine multidisciplinary discussion regarding breast cancer patients	
Weekly	29 (11%)
Biweekly	23 (8.4%)
Monthly	51 (19%)
Case-by-case basis	139 (51%)
None	31 (11%)
Unknown	4
Specialties providing breast cancer care at practice facility	
Surgeon	266 (96%)
Pathologist	244 (88%)
Radiologist	277 (82%)
Nurse	220 (79%)
Pharmacist	174 (63%)
Clinical oncologist	166 (60%)
Pathology laboratory technician	138 (50%)
Social worker	121 (44%)
Nutritionist/dietician	116 (42%)
Palliative care	103 (37%)
Radiology technologist	101 (36%)
Psychiatrist/psychologist/mental health professional	101 (36%)
Physical therapist	82 (30%)
Patient advocate/patient navigation	54 (19%)

© 2025 Romanoff A et al. *JAMA Network Open*.

eTable 2. Workflow Process

	Healthcare Providers N=277
Do you receive formal feedback about the care you provide?	
Direct patient comments	173 (62%)
Informal from supervisor/colleagues	152 (55%)
Formal from department/institution	45 (16%)
Written report	26 (9.4%)
Targeted metrics/data-driven feedback	9 (3.2%)
None/I don't receive feedback	38 (14%)
How do you typically find out about practice-changing innovations?	
Personal reading of the medical literature	224 (81%)
Interaction with colleagues	212 (77%)
Institutional meetings	178 (64%)
National conferences	176 (64%)
Professional trainings	167 (60%)
International conferences	123 (44%)
How do you connect with colleagues to discuss complex clinical cases?	
Departmental meetings	220 (79%)
Informal discussion with colleagues	186 (67%)
Instant messaging platform (including WhatsApp)	155 (56%)
Formal multidisciplinary meetings/institutional tumor board conferences	154 (56%)
National/international conferences	88 (32%)
Virtual tumor board conference (e.g., Project ECHO)	81 (29%)
Professional society online fora/listservs	68 (25%)
Social media	41 (15%)
Do you have an institutional database or registry to collect information on breast cancer patients at your center?	
Yes	147 (60%)
No	56 (23%)
I don't know	41 (17%)
Unknown	33

	**Never**	**Rarely**	**Sometimes**	**Most of the Time**	**Always**
How often do you have access to an electronic data system that allows you to track patients (e.g., electronic medical record)? (n=273)	94 (34%)	67 (25%)	51 (19%)	31 (11%)	30 (11%)
To what extent do you network with colleagues or people in similar professions/positions outside your clinical practice setting? (n=272)	6 (2.2%)	20 (7.4%)	132 (49%)	76 (28%)	38 (14%)
To what extent do you feel like you can try new things to improve your work process? (n=272)	2 (0.7%)	20 (7.4%)	86 (32%)	95 (35%)	69 (25%)
To what extent does your organization support you in trying new things or adopting	22 (8.0%)	68 (25%)	121 (44%)	50 (18%)	13 (4.7%)

© 2025 Romanoff A et al. *JAMA Network Open*.

new innovations to improve your work processes? (n=274)					
	**Not at all receptive**	**Slightly receptive**	**Somewhat receptive**	**Moderately receptive**	**Extremely receptive**
What do you think would be the level of receptivity by your organizations leadership to implementing structured breast cancer care programs to promote guideline adherence? (n=271)	10 (3.7%)	35 (13%)	68 (25%)	94 (35%)	64 (24%)

© 2025 Romanoff A et al. *JAMA Network Open*.

eFigure. Forest Plot of Multivariable Associations With Consulting Guidelines Regularly

Covariate	Level	OR (95%CI)
Profession	Surgeon	0.6 (0.23-1.54)
	Pathologist	0.16 (0.05-0.51)
	Radiologist	0.07 (0.02-0.26)
Completed fellowship in cancer care	Yes	5.91 (2.03-19.42)
Percent of practice devoted to breast healthcare		1.03 (1.01-1.05)
Engaged in multi-disciplinary discussion	Weekly	1.6 (0.39-6.8)
	Biweekly	1.89 (0.5-7.56)
	Monthly	2.0 (0.61-7.17)
	Case-by-case basis	1.81 (0.64-5.72)

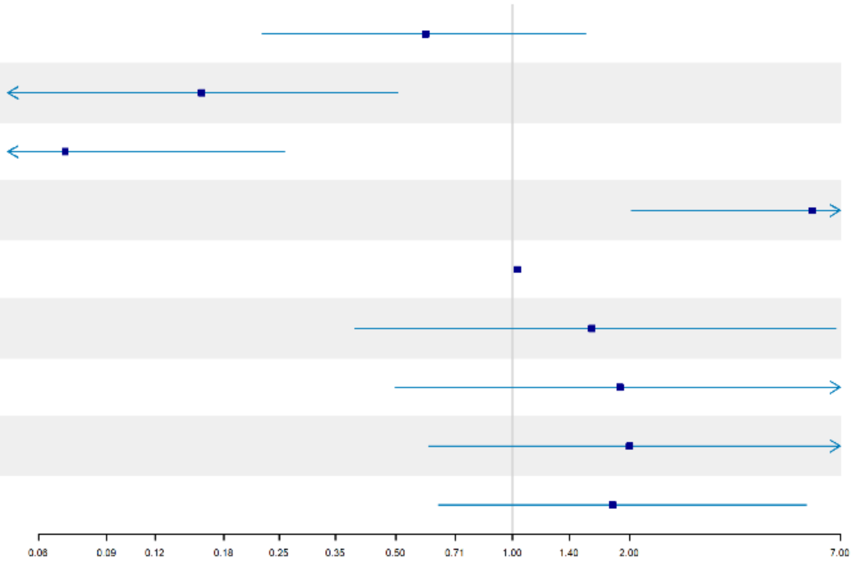

© 2025 Romanoff A et al. *JAMA Network Open*.
