# Supplementary material for: Health Care Professional Adherence to Breast Cancer Management Guidelines in Nigeria
Source: JAMA Netw Open. 2025 Feb 12;8(2):e2459614. doi: 10.1001/jamanetworkopen.2024.59614 (PMC11822546; doi:10.1001/jamanetworkopen.2024.59614)
Supplement: Supplement 2. — Data Sharing Statement [file jamanetwopen-e2459614-s002.pdf]

Data Sharing Statement

Romanoff. Health Care Professional Adherence to Breast Cancer Management Guidelines in Nigeria. *JAMA Netw Open*. Published February 12, 2025.
doi:10.1001/jamanetworkopen.2024.59614

Data
Data available: Yes
Data types: Data dictionary
How to access data: kahnr1@mskcc.org
When available: With publication

Supporting Documents
Document types: None

Additional Information
Who can access the data: Researchers whose proposed use of the data has been approved.
Types of analyses: Research purposes.
Mechanisms of data availability: After approval of a proposal
